# Gender disparities in the associations of behavioral factors, serious psychological distress and chronic diseases with type 2 diabetes screening among US adults

**DOI:** 10.1186/s41256-018-0065-z

**Published:** 2018-04-03

**Authors:** Xin Xie, Nianyang Wang, Ying Liu

**Affiliations:** 1 Department of Economics and Finance, College of Business and Technology, East Tennessee State University, PO Box 70686, 227 Sam Wilson Hall, Johnson City, TN 37614 USA; 2 Department of Preventive Medicine, Feinberg School of Medicine, Northwestern University, Chicago, IL 60611 USA; 3 Department of Biostatistics and Epidemiology, College of Public Health, East Tennessee State University, Johnson City, TN 37614 USA

**Keywords:** Type 2 diabetes screening, Prevalence, Behavioral factors, Mental health, Chronic diseases, Disparities

## Abstract

**Background:**

The increasing prevalence of undiagnosed and diagnosed type 2 diabetes (T2D) posed a major challenge for public health and thus screening for T2D becomes essentially important. The social-demographical factors associated with the use of T2D screening have been widely studied, however, little is known about the impact of behavioral factors, mental health and chronic diseases on prevalence of screening, especially by gender and age groups.

**Methods:**

We investigated the impact of behavioral factors, mental health and chronic diseases across gender and age groups on the usage rate of T2D screening. To analyze the likelihood of the use of T2D screening, we performed weighted binomial logistic regression analyses.

**Results:**

Obesity, physical activity and smoking increased the use of T2D screening for females more than for males, and alcohol use increased screenings only for females. Serious psychological distress (SPD) was found to have a positive association with the use of T2D screening for females rather than for males; whereas hypertension and diabetes increased the use of T2D screening for males more than for females. Physical activity was an effective predictor of screening for T2D in the groups of 45–64 years and 65 years or older. Former drinking was positively associated with T2D screening for people aged 65 or older, and smoking was found to increase the odds of screening for T2D for people aged less than 65.

**Conclusions:**

Behavioral factors, mental health, and chronic diseases were significantly associated with the use of T2D screening and further demonstrated that gender differences exist in the role of above factors.

## Background

The current high prevalence of both undiagnosed and diagnosed type 2 diabetes (T2D) and their rising trends worldwide became concerns in public health. A recent study estimated that the worldwide population of those with diabetes was expected to rise from 366 million in 2011 to 552 million in 2030 [1]. T2D can affect patients’ organs and thus lead to serious complications, such as hypertension, abnormal heart attack, stroke, blindness, kidney failure, and loss of feet or legs, etc. In addition, the latent phase of the condition can be as long as 9–12 years [2]. Therefore, it is not surprising that 30%–50% of all cases of T2D were undiagnosed [3]. In addition to the high prevalence of T2D, including undiagnosed cases, substantial burden was imposed by diabetes on society. The estimated total economic cost of diagnosed diabetes in 2012 was $245 billion, which was 41% higher than the previous estimate of $174 billion dollars in 2007 [4]. A long latent period, high prevalence of undiagnosed diabetes, serious outcomes of complications, and a high economic burden are strong arguments for preventive screening. T2D screening is of great importance in that it brings forward prevention, a timely diagnosis and treatment. With lifestyle intervention, people deemed at high risk of T2D can avoid developing diabetes [5]. Once diabetes develops, the likelihood of serious complications can be significantly reduced by timely diagnosis and proper medical management [6].

To promote T2D screening program, it is essential for policy makers and healthcare providers to identify targeted people who are at a high risk of developing T2D and understand factors associated with the use of T2D screening, so that they could develop appropriate recommendations for the health care community and provide effective dissemination support. There is an abundance of literature on the risk factors associated with having T2D. For example, one study indicated that many demographic, socioeconomic, behavioral risk, and health status characteristics are strongly associated with prevalence of T2D and therefore the authors believe that the screening for diabetes should be broadly focused on the whole population with selective blood testing [7]. Another study presented the negative association between social position and incidence of diabetes and this relationship is stronger in men than in women [8].

The existing extensive studies have examined the factors associated with the use of preventive care, including diabetes screening. Among the current literature, insurance status, race and gender were the factors that were most commonly studied. For example, it was found by a study that uninsured adults were less likely to use preventive screenings for high cholesterol and diabetes than insured adults [9]. Another study explored potential mediators linking race/ethnic disparities to reduced receipt of preventive care and concluded that minority, rural, low-income, uninsured, and young diabetes patients were less likely to receive diabetes preventive care [10]. One recent study found that uninsured African Americans and Hispanics have higher likelihood of receiving preventive care than uninsured Whites [11]. Gender also makes a difference in using T2D screening. It was found that men were less likely to utilize preventive care than women [12]. However, another study found no substantial socioeconomic difference for the high-risk population in attending T2D screening program [13].

Although previous studies focused on the associations of diabetes preventive screening with social-demographic and socioeconomic factors, little is known about how the use of T2D screening is influenced by behavior factors, mental health and chronic diseases and even less is known about whether this influence would vary by gender and age. Therefore, this paper aims to explore the influence of behavior factors, mental health and chronic diseases on the use of T2D screening among adults in United State (U.S.) using the data from the 2014 National Health Interview Survey (NHIS); in addition to testing whether such associations differ by gender and age.

## Methods

### Data sources

The NHIS is a multi-purpose health survey which is conducted by the National Center for Health Statistics (NCHS), Center for Disease Control and Prevention (CDC). It is a principal source of health information of the civilian noninstitutionalized household population of the U.S. The NHIS has been conducted continually since it began in 1957. Public-use data files are released annually and can be accessed from the internet. NHIS survey sample is completed based on a 50-state design with independent, multistage area probability sample for each of the 50 states and the District of Columbia. Data collection is performed in a face-to-face interviewing format. From each family in the NHIS, one adult aged 18 years or older is randomly selected to respond to sample adult core questionnaires. This study used the 2014 NHIS survey with a total household response rate of 73.8% and unconditional or final response rate of 73.1% for the family component. Details of the survey design and data collection methods are available on CDC website: https://www.cdc.gov/nchs/nhis/index.htm. The total sample size for the 2014 survey is 36,697. The current analysis was restricted to participants aged 18 and older.

### Measurements

#### Outcome variable

Participants were considered to have had a T2D screening (case) if they responded “yes” to the question “Have you had a fasting test for high blood sugar or diabetes during the past 12 months?” (Table 1). Subjects who answered “no” to the question served as controls.

**Table 1:** Type 2 diabetes screening prevalence (%)

Variable	Total (N)	Cases (N)	Prevalence (%)	95% CI	*P*-value
Gender					
Male	15,979	5082	30.6	29.6–31.6	< 0.0001
Female	19,804	7112	34.6	33.5–35.8	
Age group					
18–44	15,378	3218	20.3	19.3–21.3	< 0.0001
45–64	12,005	4789	40.3	38.9–41.6	
65 +	8400	4187	49.9	48.3–51.5	
Race					
White	27,530	9501	33.2	32.4–34.1	0.0056
AA	5024	1677	30.7	28.9–32.4	
Asian	2073	643	30.9	28.3–33.5	
Other	1156	373	29.4	25.7–33.1	
Marital status					
Married	15,699	5972	37.5	36.3–38.6	< 0.0001
Divorced	11,709	4434	35.4	34.1–36.7	
Never	8375	1788	18.5	17.2–19.7	
Education					
No	14,570	4770	30.4	29.4–31.5	< 0.0001
Yes	21,063	7378	34.2	33.2–35.2	
Insurance					
No	2092	521	22.4	20.1–24.7	< 0.0001
Yes	28,850	10,975	36.6	35.7–37.4	
Obesity					
No	23,189	6930	28.7	27.8–29.7	< 0.0001
Yes	12,594	5264	40.2	38.9–41.5	
Activity					
No	13,952	4578	31.4	30.1–32.6	0.0243
Yes	20,900	7226	33.1	32.2–34.1	
Alcohol use					
Never	7480	2435	30.6	29.0–32.1	0.0009
Past	5494	2374	42.6	40.8–44.3	
current	22,508	7303	31.4	30.5–32.4	
Smoking status					
Never	21,367	7078	31.9	31.0–32.9	< 0.0001
Past	8101	3431	40.8	39.2–42.4	
Current	6248	1663	24.7	23.1–26.3	
SPD					
No	33,934	11,509	32.6	31.8–33.4	0.005
Yes	1240	472	37.5	33.9–41.1	
Hypertension					
No	23,678	6108	24.8	23.9–25.7	< 0.0001
Yes	12,074	6079	50.3	48.9–51.7	
Diabetes					
No	31,420	8908	27.5	26.7–28.3	< 0.0001
Yes	4350	3282	75.9	74.1–77.6	
Overall	35,783	12,194	32.7	31.9–33.5	

Abbreviations: *AA*; African American; *SPD*; Serious psychological distress; *P*-value is based on χ^2^ test

#### Social-demographic variables

In this analysis included age, classified as young (18–44 years), middle aged (45–64 years), and elderly (65 years or older); gender; and race/ethnicity (White, African American (AA), Asian, and other). Other demographic characteristics included education (≤ high school, > high school) and health insurance prevalence (yes, no). Marital status was classified into married/living with partner, widowed/divorced/separated, and never married.

#### Behavioral factors

Smoking status was classified as never smoked, current smoking, or past smoking. Alcohol consumption was classified as never, current light or moderate drinking, and past drinking. Physical activity was determined by the question “Do you do light or moderate leisure-time physical activities for at least 10 minutes that causes only light sweating or a slight to moderate increase in breathing or heart rate?” (yes/no). Adult obesity was defined as a body mass index (BMI) of 30.0 kg/m2 or above, and BMI was calculated as weight in kilograms divided by height in meters squared. In order to account for racial differences in body fat percentage at the same BMI level, we examined overweight and obesity using the WHO Asian BMI cut points in Asian groups as ≥27.5 kg/m^2^ (obese) [14].

#### Chronic diseases

Hypertension was defined by the question “Have you ever been told by a doctor or other health professional that you had Hypertension, also called high blood pressure?” Diabetes was defined by the question “(If female, other than during pregnancy) Have you ever been told by a doctor or health professional that you have diabetes or sugar diabetes?”

#### Mental health

Serious psychological distress (SPD) is a nonspecific measure of psychological distress that has been psychometrically validated and shown to be able to differentiate community Diagnostic and Statistical Manual of Mental Disorders, Fourth Edition (DSM-IV) cases from noncases [15, 16]. It is intended to characterize having at least one mental disorder, such as major depressive disorder, generalized anxiety disorder, or schizophrenia, as well as having serious impairment of body function. SPD was determined using the K6 scale, which is comprised of 6 questions asking how often during the past 30 days a person felt “so sad that nothing could cheer them up,” “nervous,” “restless,” “hopeless,” “worthless,” or “everything was an effort.” Responses were scored from 0 (none of time) to 4 (all the time) and summed to produce a total score (0 to 24), with a score of 13 or above used to define SPD [15].

### Statistical analysis

Population proportions in cases and controls of independent variables and demographic factors were estimated by the PROC SURVEYFREQ procedure. The PROC SURVEYMEANS procedure was used to estimate the overall prevalence of T2D screening; while PROC SURVEYFREQ was used to estimate the prevalence in potential factors. A Chi-square test was used to compare prevalence of T2D screening across groups. Then, SAS PROC SURVEYLOGISTIC was used to estimate the odds ratios (ORs) and 95% confidence intervals (CIs) for the relation between potential factors and T2D screening. We implemented univariate logistic analysis to examine the independent roles of potential factors in the use of T2D screening. Afterwards, we applied multiple logistic regressions to simultaneously adjust for all potential factors of T2D screening. Controlling variables include gender, age, race, marital status, education, insurance, obesity, activity, alcohol use, smoking status, SPD, hypertension, and diabetes. To examine the factors for T2D screening stratified by gender and age group, a multiple logistic regression was were applied to adjust for all these factors.

All the analyses were conducted with SAS statistical software, version 9.4 (SAS Institute, Cary, NC, USA).

## Results

### Prevalence of the use of type 2 diabetes screening

Table 1 displays the prevalence of T2D screening in 2014 by demographic, social, and economic characteristics. The overall prevalence of T2D screening is 32.7%. As indicated in Table 1, gender and age make differences in the prevalence of T2D screening. In particular, females (34.6%) had a higher prevalence of utilizing T2D screening than males (30.6%) and older people (40.3% for 45–65 years and 49.9% for 65+, respectively) had higher prevalence to screen for T2D than younger people (20.3% for 18–44 years). Table 1 also shows that those who have had T2D screening were more likely to be married, obese, to have a higher income, to have health insurance, to participate in physical activity, to have past drinking and smoking experiences, and to have the diseases of SPD, hypertension, and diabetes. Chi-square tests indicate that each of these differences is statistically significant (*P* < 0.0001).

### The relationship between potential risk factors and the use of type 2 diabetes screening

Table 2 presents the results of univariate and multiple logistic regression analyses by using the full sample. Under the univariate analysis, all the factors are shown to be associated with the behavior of T2D screening (*P* < 0.05). These results are quite consistent with the ones in Table 1 because the crude odd OR shows the relation between each individual factor with T2D screening without taking into account all other factors.

**Table 2:** Univariate and multiple logistic regression analyses for the relationship between potential factors and type 2 diabetes screening

Variable	Crude OR	95% CI	*P*-value	Adjusted OR	95% CI	*P*-value
Gender						
Male	1			1		
Female	1.20	1.13–1.28	< 0.0001	1.20	1.12–1.30	< 0.0001
Age group						
18–44 years	1			1		
45–64 years	2.65	2.45–2.86	< 0.0001	1.77	1.61–1.95	< 0.0001
65 +	3.91	3.62–4.23	< 0.0001	1.83	1.64–2.05	< 0.0001
Race						
White	1			1		
AA	0.89	0.82–0.97	0.0053	0.93	0.83–1.03	0.164
Asian	0.84	0.69–1.01	0.057	0.98	0.77–1.26	0.883
Other	0.90	0.79–1.02	0.081	1.05	0.91–1.21	0.496
Marital status						
Never	1			1		
Married	2.64	2.41–2.89	< 0.0001	1.56	1.40–1.75	< 0.0001
Divoice	2.42	2.19–2.66	< 0.0001	1.33	1.18–1.50	< 0.0001
Education						
No	1			1		
Yes	1.19	1.12–1.26	< 0.0001	1.28	1.18–1.38	< 0.0001
Insurance						
No	1			1		
Yes	1.99	1.74–2.29	< 0.0001	1.58	1.36–1.84	< 0.0001
Obesity						
No	1			1		
Yes	1.65	1.53–1.77	< 0.0001	1.25	1.16–1.35	< 0.0001
Activity						
No	1			1		
Yes	1.08	1.01–1.16	0.0245	1.19	1.09–1.30	< 0.0001
Alcohol use						
Never	1			1		
Past	1.68	1.53–1.85	< 0.0001	1.17	1.03–1.33	0.0137
Current	1.04	0.96–1.13	0.329	1.12	1.01–1.24	0.0375
Smoking status						
Never	1			1		
Past	1.44	1.31–1.57	< 0.0001	1.32	1.16–1.50	< 0.0001
Current	2.10	1.90–2.33	< 0.0001	1.32	1.18–1.48	< 0.0001
SPD						
No	1			1		
Yes	1.25	1.07–1.45	0.005	1.06	0.86–1.31	0.571
Hypertension						
No	1			1		
Yes	3.07	2.87–3.28	< 0.0001	1.70	1.55–1.85	< 0.0001
Diabetes						
No	1			1		
Yes	8.29	7.50–9.15	< 0.0001	5.72	5.08–6.44	< 0.0001

Abbreviations: *AA*; African American; *SPD*; Serious psychological distress; *OR*; Odds ratio; CI = Confidence interval

After adjusting for other factors, females (OR = 1.20, 95% CI = 1.12–1.30) and older age groups (OR = 1.77, 95% CI = 1.61–1.95 for the age group 45–64, OR = 1.83, 95% CI = 1.64–2.04 for the group 65+) were positively associated with T2D screening. When comparing behavior factors: obesity, participation in physical activity, and smoking experience were positively related with the use of T2D screening while drinking was not. As to chronic diseases and mental health, while SPD was not shown to be related with T2D screening, hypertension and diabetes were positively associated with T2D screening.

To further explore the association of the above factors with the use of T2D screening, we conducted additional analysis to predict the association between factors and using T2D screening across gender and age groups.

### Gender differences in the association between potential factors and type 2 diabetes screening

Table 3 displays multiple logistic regression results for the relationship between potential factors and the use of T2D screening across gender groups. Several social-demographic factors emerged as significant predictors for both males and females. The groups aged 45–64 and greater than 65 are more likely to go through T2D screening test than their counterpart group aged 18–44 for both genders. Marital status also predicted screening for T2D. In regards to behavioral factors: obesity, participation in physical activity, and smoking were associated with higher probability of screening for T2D for both males and females, but these associations were stronger for females. Past drinking was more likely to be correlated with the use of screening for T2D just for females. Current drinking was not correlated for either gender. Among mental health and chronic diseases, males with SPD had a higher likelihood to screen for T2D, but we found no such evidence for females who had SPD. Hypertension and diabetes were positively associated with T2D screening for both genders, and a stronger association was found among males.

**Table 3:** Multiple logistic regression analysis for the relationship between potential factors and type 2 diabetes screening by gender

Variable	OR^a^	95% CI	*P*-value	OR^b^	95% CI	*P*-value
Age group						
18–44 years	1			1		
45–64 years	1.92	1.67–2.21	< 0.0001	1.66	1.46–1.88	< 0.0001
65 +	2.01	1.70–2.36	< 0.0001	1.72	1.49–1.99	< 0.0001
Race						
White	1			1		
AA	0.90	0.74–1.09	0.291	0.94	0.82–1.08	0.384
Asian	1.00	0.69–1.46	0.982	0.94	0.69–1.27	0.667
Other	1.15	0.92–1.44	0.233	0.98	0.80–1.20	0.855
Marital status						
Never	1			1		
Married	1.67	1.42–1.97	< 0.0001	1.48	1.28–1.71	< 0.0001
Divoice	1.37	1.15–1.63	0.0004	1.33	1.13–1.58	0.0007
Education						
No	1			1		
Yes	1.33	1.18–1.51	< 0.0001	1.21	1.09–1.34	0.0003
Insurance						
No	1			1		
Yes	1.78	1.37–2.31	< 0.0001	1.47	1.21–1.79	< 0.0001
Obesity						
No	1			1		
Yes	1.17	1.04–1.33	0.0118	1.33	1.21–1.46	0.0001
Activity						
No	1			1		
Yes	1.15	1.01–1.32	0.0476	1.22	1.10–1.36	0.0002
Alcohol use						
Never	1			1		
Past	1.04	0.85–1.27	0.719	1.23	1.05–1.45	0.0109
Current	1.05	0.90–1.24	0.518	1.13	1.00–1.29	0.0636
Smoking status						
Never	1			1		
Past	1.28	1.05–1.57	0.0157	1.36	1.14–1.61	0.0006
Currrent	1.32	1.10–1.57	0.0023	1.35	1.15–1.57	0.0002
SPD						
No	1			1		
Yes	1.58	1.06–2.37	0.0264	0.84	0.65–1.08	0.168
Hypertension						
No	1			1		
Yes	1.96	1.74–2.21	< 0.0001	1.49	1.32–1.67	< 0.0001
Diabetes						
No	1			1		
Yes	6.07	5.11–7.20	< 0.0001	5.32	4.49–6.29	< 0.0001

^a^Male; ^b^Female

Abbreviations: *AA*; African American; *SPD*; Serious psychological distress; *OR*; Odds ratio; CI = Confidence interval

### Age differences in the association between potential factors and type 2 diabetes screening

Table 4 summarizes the results from multiple logistic regressions that predict the use of T2D screening across age groups. One interesting finding is that young females were more likely to use T2D screening than their male counterparts (OR = 1.41, 95% CI =1.23–1.61) for the age group 16–44 years, but gender did not make any difference in screening T2D in the other two age groups. Among the three age groups, race was only found to be associated with the use of T2D screening in adults aged 45 years old or over. In particular, African Americans were less likely to screen for T2D than Whites in the age group 45–64 years, and Asians were more likely than Whites to go for screening test for T2D in the age group 65 years or older. The levels of behavior disparities in T2D screening were also different by age. Obesity was associated with a higher probability with T2D screening in the highest and lowest age groups. Participation in physical activity was also a predictor of screening for T2D in the group of 45–64 years and the group of 65 or older. Past drinking was positively associated with T2D screening for people aged 65 or older, and smoking was found to increase the odds of screening for T2D for people aged less than 65. Hypertension and diabetes significantly increased the likelihood of screening for T2D, but no relation between SPD and T2D screening was found in any age groups.

**Table 4:** Multiple logistic regression analysis for the relationship between potential factors and type 2 diabetes screening by age group

Variable	OR^a^	95% CI	*P*-value	OR^b^	95% CI	*P*-value	OR^c^	95% CI	*P*-value
Sex									
Male	1			1			1		
Female	1.41	1.23–1.61	< 0.0001	1.12	0.99–1.27	0.0608	1.04	0.90–1.20	0.624
Race									
White	1			1			1		
AA	1.08	0.90–1.29	0.412	0.80	0.67–0.95	0.0131	0.80	0.66–0.97	0.0205
Asian	0.93	0.66–1.32	0.695	0.80	0.51–1.25	0.320	1.81	1.15–2.87	0.011
Other	1.07	0.85–1.34	0.583	0.91	0.69–1.22	0.509	1.37	1.05–1.80	0.0226
Marital status									
Never	1			1			1		
Married	1.75	1.50–2.04	< 0.0001	1.30	1.08–1.57	0.0051	1.17	0.86–1.58	0.326
Divoice	1.34	1.09–1.65	0.0063	1.12	0.92–1.37	0.245	1.17	0.88–1.57	0.282
Education									
No	1			1			1		
Yes	1.22	1.06–1.41	0.0062	1.29	1.14–1.47	< 0.0001	1.27	1.12–1.44	0.0003
Insurance									
No	1			1			1		
Yes	1.39	1.12–1.73	0.0028	2.02	1.62–2.52	< 0.0001	1.00	0.57–1.77	0.998
Obesity									
No	1			1			1		
Yes	1.43	1.26–1.62	< 0.0001	1.12	0.98–1.27	0.0846	1.18	1.02–1.35	0.0247
Activity									
No	1			1			1		
Yes	1.13	0.97–1.30	0.116	1.17	1.04–1.32	0.0104	1.30	1.14–1.50	0.0002
Alcohol use									
Never	1			1			1		
Past	1.23	0.94–1.61	0.137	1.12	0.90–1.39	0.311	1.19	0.98–1.45	0.0724
Current	1.03	0.87–1.23	0.738	1.12	0.94–1.34	0.218	1.23	1.03–1.46	0.0229
Smoking status									
Never	1			1			1		
Past	1.30	1.04–1.62	0.0202	1.38	1.12–1.70	0.0025	1.24	0.97–1.58	0.0889
Current	1.32	1.11–1.58	0.0022	1.41	1.17–1.70	0.0003	1.23	0.96–1.58	0.101
SPD									
No	1			1			1		
Yes	1.19	0.83–1.70	0.337	0.93	0.68–1.27	0.643	1.06	0.68–1.66	0.783
Hypertension									
No	1			1			1		
Yes	2.08	1.73–2.50	< 0.0001	1.71	1.50–1.94	< 0.0001	1.42	1.23–1.63	< 0.0001
Diabetes									
No	1			1			1		
Yes	7.12	5.14–9.85	< 0.0001	6.43	5.28–7.83	< 0.0001	4.92	4.12–5.87	< 0.0001

^a^18–44 years,; ^b^45–64 years; ^c^65 or older

Abbreviations: *AA*; African American; *SPD*; Serious psychological distress; *OR*; Odds ratio; CI = Confidence interval

## Discussion

This study examines the influence of potential factors with a particular focus on behavioral factors, mental health and chronic diseases on the use of T2D screening and further explores gender and age differences in this influence. Gender differences measure how the influence of potential factors on T2D screening varies by gender, while age difference is the variation of the influence of potential factors on T2D screening across age groups. We found that gender, age, marital status, education level, insurance status, obesity, participation in physical activity, alcohol use, smoking, SPD, hypertension, and diabetes are all associated with the use of T2D screening. After adjusting for covariates, all above factors are still significantly associated with the use of T2D screening except for SPD. In addition, gender and age differences exist in associations of the behavioral factors, mental health and chronic diseases with the use of T2D screening.

The overall prevalence of the use of T2D screening is 32.7% in U.S. adults, which suggests that the concept of diabetes screening has not yet become the norm. This study provided evidence that females were more likely than males to use T2D screening overall. This result could be partially explained by the greater influence of diabetes for females than their male counterparts. A previous study found that females were more related with a negative impact of diabetes on daily life than males [17]. However, this influence only held for the age group 18–44, which implies no gender difference of T2D screening for older people. A very possible reason for the age difference of association between gender and T2D screening is that females in fertility age (18–44 years) are more likely to have T2D screenings than those in other age groups. Our study also found that the older age groups were more associated with the use of T2D screening, which is understandable because T2D prevalence increased with age [18]. It is common that older people are more likely to suffer from the sickness and care about their health status. In addition, older age groups have a higher likelihood to have health insurance coverage [19]. Free screening services provided by health insurance coverage could potentially explain why elderly people are more likely to use T2D screening.

Our findings showed that behavioral factors such as obesity, physical activity, alcohol use, and smoking status were all associated with a higher probability of the use of T2D screening. Even though a previous study showed that obese patients were significantly less likely to receive various cancer screenings [20], our results showed that the obese population was more likely to use T2D screening. This can be explained by the finding that most adults with diagnosed diabetes were overweight or obese [21]. Our results also demonstrated that this effect was stronger for females than for males, and for the young than for the older, which implies females and young people were more concerned about the possibility that obesity induces T2D.

In regards to physical activity, it was reported that a higher diabetes risk was more likely to go to people with brisk and higher levels of exercise physical activity [22]. Combined with our result on the association of physical activity with the use of T2D screening, we might understand physical activity as the awareness to health status and the stronger association between physical activity and the use of T2D screening in women implies that females had more awareness of health care than males. Similarly, the age difference in the influence of physical activity on using T2D screening suggests elderly people are more health-conscious.

Alcohol use was only shown to increase the probability of using T2D screening in women but not in men, and smoking was associated with the use of T2D screening for females more than for their male counterparts. The literature consistently reports that men drink alcohol and smoke more frequently and at a greater amount than women [12] and that the prevalence of alcohol abuse and smoking were significantly higher in men than in women [23]. The gender differences in substance-related high risk behaviors were explained by traditional masculinity [24]. This could also indirectly explain the difference in using T2D screening between genders. Males and females’ attitude to drinking alcohol and smoking might affect their health preventive behavior. For males, substance use were viewed as a masculine behavior and therefore normal, so they might not think this is a high risk behavior as much as females do and therefore are less likely than females to use T2D screening. The way people’s attitudes toward substance use affect their preventive behaviors also varied by age. The age difference in this respect suggested that only current alcohol users age 65 or older viewed drinking alcohol as a T2D causing behavior and that smokers age 65 or older did not think there was a connection between smoking and T2D.

Even though many research studies have found an association between diabetes and an increased risk for depression [25–28], our results did not show that SPD was associated with the use of T2D screening overall when adjusting for other covariates. This is very likely due to the high correlation between SPD and some other variables, such as diabetes. Only the gender difference was found in the influence of SPD on using T2D screening. In particular, SPD increased the odds ratio of using T2D screening in men but decreased that in women. The gender difference in the behavior of T2D screening for those diagnosed with SPD could be attributed to the gender difference in mental health. The evidence suggests that different types of mental disorders for men and women are the partial reasons why women are more likely to have chronic debilitating conditions while men are more likely to have life threatening conditions [29]. Therefore, it is likely that T2D tends to cause complications that are life threatening to males but chronic debilitating to females due to SPD.

Chronic diseases were positively related with the use of T2D screening in our study. It was reported that local health departments which conduct chronic disease surveillance was significantly associated with conducting diabetes screening programs [30]. It is well-known that hypertension is significantly associated with the likelihood of T2D in the overall population and among the complications of T2D. Therefore, it was not surprising that people diagnosed with hypertension would be more likely to utilize T2D screening. It was interesting to find that a diagnosis of either hypertension or diabetes increased the odds of using T2D screening more for males than for females and more for young people than older people. One explanation for the gender difference is the attitude disparity for men and women in various aspects. Research provides evidence that attitudes toward success and perceived behavioral control had effects on efforts to regulate hypertension in men but not in women, but perceived subjective norms had effects on efforts only in women [31]. The age difference could be explained in the way that chronic diseases which are not normal for the young act as a stronger warning sign to care about health, compared for the elderly.

This study has several important strengths. First, NHIS data is a principal source of health information of the civilian noninstitutionalized household population of the U.S., with a large sample size of subjects that was selected at random. The large sample size also gives us a statistical power in our estimates. Second, we provide the national prevalence estimates for T2D screening using a nationally representative sample of U.S. adults. Third, we explored how effects of behavioral factors, mental health and chronic diseases on the use of T2D screening and whether the associations differ by gender and age groups. There are several limitations as well. First, pregnancy of females may be a confounder for T2D screening; however, there is no such information in our data. Second, the cross-sectional data could not determine a temporal or causal relationship between potential factors and the use of T2D screening. In addition, self-reported data from interviews may be subjected to response bias.

## Conclusions

In summary, this study revealed that behavioral factors, mental health, and chronic diseases were significantly associated with the use of T2D screening and further demonstrated that gender differences exist in the role of the above factors as sources that are associated with the use of T2D screening. Our findings provide insightful perspectives with respect to the behavior of using T2D screening, which would be helpful for policy makers and related health organizations to design T2D intervention programs. Further research is needed to more fully uncover the reasons for the associations and the gender differences in the associations.

## Abbreviations

AA: African American
BMI: Body mass index
CI: Confidence interval
IRB: Institution Review Board
OR: Odds ratio
SPD: Serious psychological distress
T2D: Type 2 diabetes
